# Quorum Sensing and Quorum Quenching in Periodontal Disease: Mechanisms and Therapeutic Potential

**DOI:** 10.3390/cimb48060574

**Published:** 2026-05-29

**Authors:** Nada Tawfig Hashim, Rasha Babiker, Muhammed Mustahsen Rahman, Riham Mohammed, Vivek Padmanabhan, Md Sofiqul Islam, Nallan C. S. K. Chaitanya, Bakri Gobara, Shadi El Bahra

**Affiliations:** 1 Department of Periodontics, RAK College of Dental Sciences, RAK Medical & Health Sciences University, Ras Al-Khaimah 12973, United Arab Emirates; mustahsen@rakmhsu.ac.ae; 2 Department of Oral Rehabilitation, Faculty of Dentistry, University of Khartoum, Khartoum 11115, Sudan; bakrigobara10@gmail.com; 3 Department of Physiology, RAK College of Medical Sciences, RAK Medical & Health Sciences University, Ras Al-Khaimah 11172, United Arab Emirates; rashababiker@rakmhsu.ac.ae; 4 Department of Oral and Maxillofacial, RAK College of Dental Sciences, RAK Medical & Health Sciences University, Ras Al-Khaimah 12973, United Arab Emirates; riham.abdelraouf@rakmhsu.ac.ae; 5 Department of Pediatric and Preventive Dentistry, RAK College of Dental Sciences, RAK Medical & Health Sciences University, Ras Al-Khaimah 12973, United Arab Emirates; vivek.padmanabhan@rakmhsu.ac.ae; 6 Department of Operative Dentistry, RAK College of Dental Sciences, RAK Medical & Health Sciences University, Ras Al-Khaimah 12973, United Arab Emirates; sofiqul.islam@rakmhsu.ac.ae; 7 Department of Oral Radiology, RAK College of Dental Sciences, RAK Medical & Health Sciences University, Ras Al-Khaimah 12973, United Arab Emirates; krishna.chytanya@rakmhsu.ac.ae; 8 Clinical Sciences Department, College of Dentistry, Ajman University, Ajman P.O. Box 346, United Arab Emirates; s.elbahra@ajman.ac.ae

**Keywords:** quorum sensing, quorum quenching, periodontal disease, biofilm, periodontal pathogens, quorum-sensing inhibitors

## Abstract

Periodontal disease is a chronic inflammatory condition driven by polymicrobial biofilms whose interaction with the host immune response drives the destruction of tooth-supporting tissues. Within these communities, bacterial cell–cell communication—particularly quorum sensing (QS)—coordinates virulence factor expression, biofilm maturation, and interspecies behaviour, allowing pathogens to mount population-dependent attacks on the host. Disrupting these signals has therefore drawn growing attention as an anti-virulence strategy for biofilm-associated oral infection. Quorum quenching (QQ)—the inhibition or disruption of QS pathways—prevents bacteria from coordinating these virulence-related activities. The candidate inhibitors investigated to date fall into three broad classes: conventional antibiotics used at sub-inhibitory concentrations, plant-derived natural compounds, and synthetic molecules designed to interfere with signal synthesis, signal reception, or signal transduction. In experimental work on periodontal pathogens, agents from each class reduce biofilm formation, suppress virulence factor production, and disrupt microbial communication within polymicrobial biofilms. Clinical translation, however, lags behind the laboratory evidence. Most data still come from in vitro systems and animal models, and the ecological complexity of the oral biofilm makes therapeutic targeting difficult: signals that drive virulence in pathogens also support cooperation among commensals. Toxicity profiles, pharmacokinetics, and well-powered clinical trials are needed before quorum-quenching agents can be considered for routine periodontal care. Even with these caveats, targeting bacterial communication offers a different therapeutic logic from conventional antimicrobials: attenuating virulence rather than killing cells, and so exerting weaker selective pressure for resistance. Further dissection of QS networks in oral biofilms—and the rational design of quenching agents that act on pathogenic rather than commensal signalling—may yield useful adjuncts to current periodontal therapy.

## 1. Clinical Significance

Quorum sensing (QS) coordinates the bacterial communication and virulence behaviour that drive periodontal disease. Interfering with QS represents an anti-virulence strategy that may complement conventional periodontal therapy: by silencing the signals that periodontal pathogens use to synchronise their attack, it may be possible to lower their effective pathogenicity without applying the selective pressure that comes with broad-spectrum antimicrobials. This approach holds the prospect of improving treatment outcomes and slowing disease progression as part of more individualised periodontal care.

## 2. Introduction

Dental plaque is the principal etiological factor in periodontal disease [1]. It is a highly organised microbial biofilm in which diverse microorganisms adhere to the tooth surface within a self-produced extracellular polymeric matrix. The matrix—polysaccharides, proteins, nucleic acids, and lipids—provides structural stability and supports metabolic cooperation among community members [2]. Its three-dimensional architecture—with spatial organisation, microbial diversity, and functional heterogeneity—accounts for the resilience and pathogenic potential of dental plaque [3]. When plaque accumulates on the tooth surface, gingival inflammation follows; in susceptible individuals, untreated gingivitis can progress to destructive periodontitis [3,4].

Plaque begins to form within 4–12 h of cleaning. Initial colonisation proceeds through adsorption of salivary glycoproteins and other host-derived molecules onto enamel, generating the acquired pellicle [5]. The pellicle exposes receptor sites that allow early-colonising bacteria, predominantly Gram-positive facultative species, to attach. These pioneer organisms create a scaffold for sequential attachment of additional species via coaggregation and metabolic cross-feeding, ultimately producing a mature multispecies community. Late colonisers—including the principal periodontal pathogens—integrate into the established community and shift its pathogenic potential [6,7,8].

**Figure 1:**
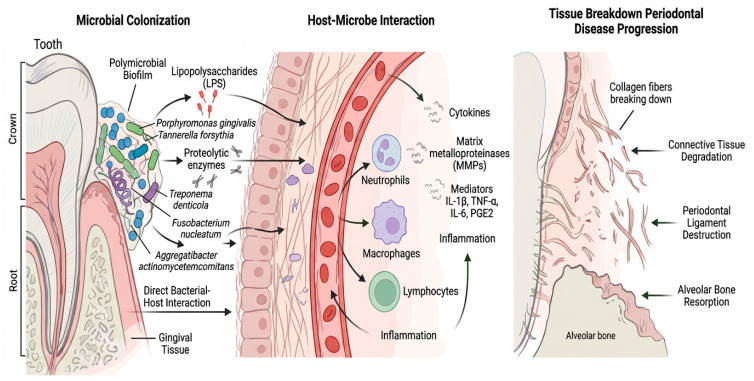
Schematic illustration of microbial–host interactions in periodontal disease progression. Left panel (Microbial Colonisation): early colonisers (*Streptococcus* spp.) attach to the acquired pellicle on the tooth surface and form supragingival biofilm; late colonisers including *Porphyromonas gingivalis*, Treponema denticola, and *Fusobacterium nucleatum* establish the subgingival biofilm within the periodontal pocket. Bacteria in the biofilm interact directly with host epithelial cells, triggering localised inflammation (indicated by a unidirectional arrow). Middle panel (Inflammation): virulence factors (LPS, gingipains, fimbriae, leukotoxin) activate host pattern-recognition receptors, recruiting neutrophils, macrophages, and T lymphocytes. Mediators released include pro-inflammatory cytokines (IL-1β, TNF-α, IL-6, IL-8), matrix metalloproteinases (MMPs), and prostaglandins. Right panel (Tissue Destruction): sustained mediator release drives RANKL-mediated osteoclastogenesis, periodontal ligament degradation, and clinical attachment loss. Figure created by the authors using the FigureLab AI web-based platform (https://chat.figurelabs.ai).

The transition from microbial colonisation to periodontal tissue destruction depends on host–microbe interactions that link the biofilm to a destructive inflammatory cascade. Bacterial lipopolysaccharide, proteolytic enzymes, and other virulence factors engage host pattern-recognition pathways, recruiting inflammatory cells and triggering release of cytokines, matrix metalloproteinases, and additional mediators of tissue breakdown [9,10]. Periodontitis is therefore best understood as dysregulated cross-talk between a polymicrobial biofilm and the host immune system, rather than as the work of any single pathogen. Sustained microbial challenge superimposed on an exaggerated inflammatory response drives the connective tissue degradation, periodontal ligament loss, and alveolar bone resorption that define the disease [1,11] (Figure 1).

Within these polymicrobial communities, coordinated behaviour is the rule rather than the exception. The principal regulatory mechanism is quorum sensing—an intercellular signalling process by which bacteria detect their population density and collectively adjust gene expression [12,13]. By synthesising, releasing, and detecting small signalling molecules called autoinducers, bacterial populations synchronise the expression of genes that govern virulence factor production, biofilm maturation, metabolic cooperation, and environmental adaptation. In periodontal biofilms this allows pathogens to gauge their numbers and to release virulence-related cargo only when the population is large enough to overwhelm host defences [14].

### 2.1. Quorum Sensing Mechanisms in Periodontal Disease

Quorum sensing (QS) is the bacterial cell–cell communication system that enables microorganisms to coordinate behaviour according to population density [12]. Bacteria synthesise, release, and detect autoinducers, which accumulate in the surrounding environment in proportion to cell number [12,13,14]. Once they exceed a threshold concentration, autoinducers bind their cognate receptors and switch on coordinated gene expression across the population. This density-dependent regulation allows bacteria to synchronise activities that would be wasteful for an individual cell—biofilm formation, virulence factor production, metabolic cooperation, and resistance to environmental stress [15,16].

QS was first described in Vibrio fischeri, in which bioluminescence is activated only when cell density crosses a critical threshold. Comparable systems have since been characterised across Gram-negative and Gram-positive bacteria and in mixed-species communities. The molecular machinery differs widely between organisms, but the core logic is conserved: bacteria sense their own density through diffusible signalling molecules and use that information to fine-tune the genes that govern survival and pathogenicity [17,18].

**Figure 2:**
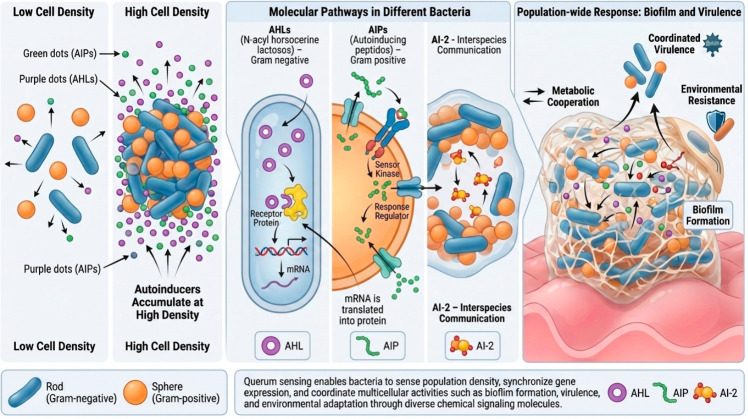
Overview of quorum sensing (QS) mechanisms in Gram-negative and Gram-positive bacteria and their role in coordinated biofilm behaviour. At low cell density, signalling molecules remain below the activation threshold, whereas at high cell density autoinducers accumulate and activate QS-regulated gene expression. Green dots represent autoinducing peptides (AIPs), the characteristic QS signals of Gram-positive bacteria, while purple dots indicate N-acyl homoserine lactones (AHLs), the principal QS molecules of Gram-negative bacteria. Orange molecules represent autoinducer-2 (AI-2), the LuxS-derived universal interspecies communication signal. Rod-shaped bacteria indicate Gram-negative species, whereas spherical bacteria represent Gram-positive species. In Gram-negative bacteria, AHLs diffuse into the cytoplasm and bind LuxR-type transcriptional regulators (yellow structure), triggering transcription of QS-responsive genes; the resulting mRNA is translated into virulence-associated proteins. In Gram-positive bacteria, AIPs are detected by membrane-bound histidine kinase (HK) sensors, which phosphorylate intracellular response regulators (RR), constituting the classical two-component signalling system that activates QS-associated genes. Collectively, these signalling pathways coordinate population-wide activities including biofilm formation, metabolic cooperation, environmental adaptation, and coordinated virulence. Figure created by the authors using the FigureLab AI web-based platform (https://chat.figurelabs.ai).

Three principal classes of QS signal have been characterised. Gram-negative bacteria typically use N-acyl homoserine lactones (AHLs), which diffuse freely across the membrane and engage intracellular transcriptional regulators. Gram-positive bacteria more often rely on autoinducing peptides (AIPs), secreted signals detected by membrane-bound two-component systems. The third class, autoinducer-2 (AI-2), is broadly distributed and functions as a cross-species signal that supports interspecies communication [13]. AI-2 has particular relevance to the oral cavity, where dozens of species coexist within structured biofilms [19]. Within these oral communities, QS controls processes that determine how the biofilm assembles and persists: microbial adhesion, extracellular matrix production, nutrient acquisition, and cooperative interactions between species. Coordinated through these signals, bacterial populations function as organised communities rather than independent cells, with corresponding gains in their ability to colonise host tissues and evade host defences [20] (Figure 2). Taken together, these three signal classes—AHLs, AIPs, and AI-2—allow the periodontal microbiome to function as a coordinated community rather than a collection of independent organisms, which is why disrupting QS is a more precise therapeutic target than broad-spectrum antimicrobial agents.

**Figure 3:**
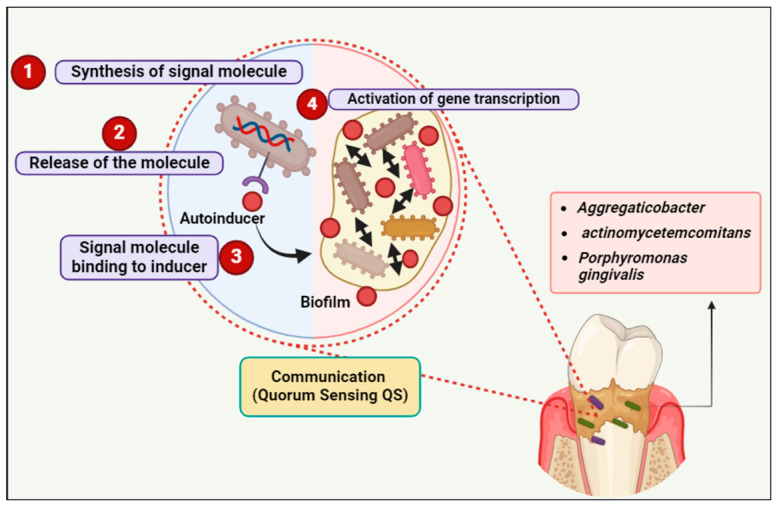
Sequential flow diagram of quorum sensing stages in periodontal pathogens (revised to a diagram format consistent with Figure 2). Stage 1—Signal Production: bacteria synthesise and secrete autoinducers (AHLs or AIPs). Stage 2—Signal Accumulation: extracellular autoinducer concentration rises as the population grows. Stage 3—Receptor Binding and Intracellular Signalling: at threshold concentration, autoinducers bind cognate receptors and activate downstream signalling cascades. Stage 4—Coordinated Gene Expression: synchronised virulence factor production across the community. Representative periodontal pathogens: *Aggregatibacter actinomycetemcomitans* (species-specific QS driving RTX leukotoxin upregulation); *Porphyromonas gingivalis* (LuxS/AI-2 regulating gingipains and FimA); *Fusobacterium nucleatum* (AI-2 interspecies bridging). Double-headed black arrow indicate the direction of signalling flow between stages [23].

In periodontal biofilms, QS regulates virulence-associated traits in several oral organisms—biofilm maturation, expression of proteolytic enzymes, and the interspecies interactions that underpin dysbiosis. Because these signals are density-dependent, periodontal pathogens can hold virulence factor expression in reserve until their population is large enough to overcome host defences [20,21]. QS therefore links the dynamics of the bacterial population to its pathogenic output, providing a layer of regulation that operates above the level of any single cell [16]. QS does more than regulate the behaviour of individual bacteria; it shapes the structure and function of entire microbial communities. In polymicrobial biofilms such as dental plaque, signalling between species influences microbial succession, cooperative metabolism, and competition for niche space [22,23]. These observations place QS at the centre of the ecological balance of the oral biofilm and identify it as a determinant of periodontal pathogenesis (Figure 3).

### 2.2. Role of Quorum Sensing in Virulence Regulation of Periodontal Pathogens

Section 2.1 outlines how QS signals accumulate and trigger coordinated gene expression. At the functional level, this makes QS a master switch for virulence: pathogens use it to hold costly virulence factor production in reserve until their population is large enough for a coordinated attack. It governs the synchronised expression of factors that drive colonisation, immune evasion, and tissue damage in medically important pathogens [15,24], allowing them to release virulence cargo only once their numbers are high enough to overwhelm host defences. Virulence factors themselves—molecules that promote host invasion, modulate the immune response, and support infection—are characteristically deployed under QS control [15]. Among QS-regulated phenotypes, biofilm formation is the most clinically consequential. Biofilms are organised microbial communities encased in a self-produced extracellular matrix that provides structural stability and shields cells from environmental stress. Within them, QS coordinates the production of matrix components, adhesion molecules, and virulence-associated proteins, supporting persistence in the face of host defences and antimicrobials while sustaining metabolic cooperation across community members [25,26].

This density-dependent control is metabolically economical: energetically expensive processes—virulence factor production and biofilm maturation—are suppressed until the population reaches a size capable of mounting a productive attack [14,15]. By acting collectively rather than as isolated cells, bacteria raise the efficiency of infection [13]. From an evolutionary standpoint, QS underpins cooperative behaviour within microbial populations—coordinated gene expression, metabolic specialisation, and community-level adaptation to environmental stress [27].

Several periodontal pathogens use QS to regulate virulence. *Aggregatibacter actinomycetemcomitans* (*A. actinomycetemcomitans*), a key organism in aggressive forms of periodontitis, upregulates RTX-family leukotoxin in response to species-specific QS signals. As the population expands, accumulating autoinducers drive transcriptional changes that raise virulence determinant expression and trigger phenotypic shifts associated with greater pathogenicity [28].

Among interspecies signals in dental plaque, autoinducer-2 (AI-2) is particularly important. AI-2 is produced through the LuxS pathway and accumulates as cell density rises [29]. Its activity has been linked to enhanced biofilm formation in oral organisms such as *Streptococcus mutans* (*S. mutans*) and the major periodontopathogens *Porphyromonas gingivalis* (*P. gingivalis*) and *Fusobacterium nucleatum* (*F. nucleatum*) [30,31]. Through this shared signal, different species in dental plaque exchange information and coordinate the behaviours that drive biofilm maturation and community stability.

**Figure 4:**
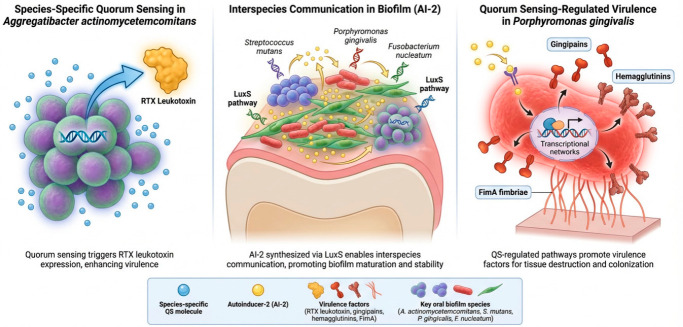
Schematic of quorum sensing in the polymicrobial periodontal biofilm. Left-most panel: blue dots = AHL autoinducers from *Aggregatibacter actinomycetemcomitans*, regulating RTX leukotoxin expression; purple circles = AI-2 molecules mediating interspecies communication. Middle panels: species-specific *A. actinomycetemcomitans* QS (left) and AI-2-mediated coordination among *P. gingivalis* and *F. nucleatum* (right). Right-most panel: yellow dots = accumulated AI-2 signal at population threshold; the shaded extracellular matrix region represents the extracellular polymeric substance (EPS), within which coordinated virulence expression and immune evasion are activated. Figure created by the authors using the FigureLab AI web-based platform (https://chat.figurelabs.ai).

In *P. gingivalis*, the LuxS/AI-2 system contributes to biofilm development and influences the expression of major virulence factors implicated in periodontal tissue destruction, including gingipains and haemagglutinins. The fimbrial protein FimA, which mediates attachment and colonisation of host tissues, sits within a transcriptional network that intersects with QS signalling [21,32] (Figure 4).

The full QS signalling networks of periodontal pathogens are not yet completely mapped, but the evidence base for their involvement in biofilm development and infection is now substantial. QS-mediated interactions support both virulence regulation and the polymicrobial synergy that drives periodontal dysbiosis, allowing different species to coordinate the metabolic and pathogenic activities that culminate in tissue destruction [33,34].

Because QS sits upstream of so much of what makes periodontal pathogens harmful, the signalling pathways themselves have become therapeutic targets. Inhibiting QS attenuates virulence without killing bacteria and therefore exerts less selective pressure for resistance than conventional antimicrobials. Strategies aimed at interrupting QS-regulated processes—biofilm maturation, virulence factor expression, and interspecies communication—are being explored as adjuncts to standard periodontal therapy [35,36].

QS in periodontal pathogens was first described in studies of *P. gingivalis* biofilm development, in which coordinated gene expression was shown to depend on AI-2. The LuxS enzyme responsible for AI-2 synthesis was subsequently found to be widely distributed among periodontal bacteria, and AI-2 signalling shown to coordinate biofilm formation and virulence factor expression in several of them [21]. These observations established QS as a candidate target for anti-virulence strategies against periodontal infection.

### 2.3. Quorum Sensing as a Modulator of Host Immune Signalling in Periodontal Disease

QS has traditionally been framed as a way for bacteria to talk among themselves. Growing evidence shows, however, that QS molecules interact directly with host immune pathways, acting as modulators of host–microbe cross-talk rather than purely as intra- or interspecies bacterial signals [24,32]. On this view, QS is not only a regulator of microbial behaviours but a chemical bridge between microbial dysbiosis and the host inflammatory response.

**Figure 5:**
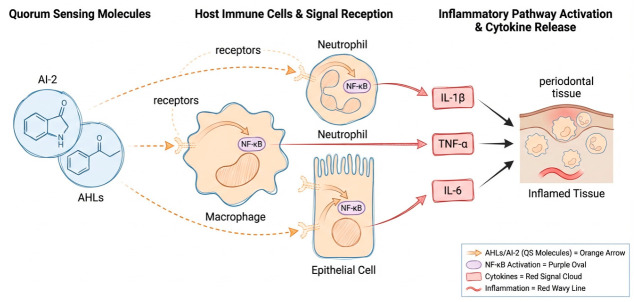
Quorum sensing–driven activation of host inflammatory responses in periodontal tissues. Left panel: QS molecules (AI-2 and AHLs) bind receptors on macrophages, neutrophils, and epithelial cells, activating NF-κB. Middle panel (“Cytokine Release”): NF-κB activation drives secretion of IL-1β, TNF-α, and IL-6 from immune and epithelial cells. Right panel (“Inflammatory Pathway Activation”): downstream signalling promotes osteoclastogenesis, MMP secretion, and progressive periodontal tissue breakdown including alveolar bone loss and clinical attachment loss. Figure created by the authors using the FigureLab AI web-based platform (https://chat.figurelabs.ai).

Both AI-2 and N-acyl homoserine lactones (AHLs) engage host cell signalling. They can interact with macrophages, neutrophils, and epithelial cells, and through these interactions modulate inflammatory pathways. In particular, QS signals activate nuclear factor-κB (NF-κB), the central transcription factor for pro-inflammatory cytokines such as IL-1β, TNF-α, and IL-6. By feeding into NF-κB, QS molecules amplify and prolong the inflammatory response within periodontal tissues [37,38] (Figure 5).

**Figure 6:**
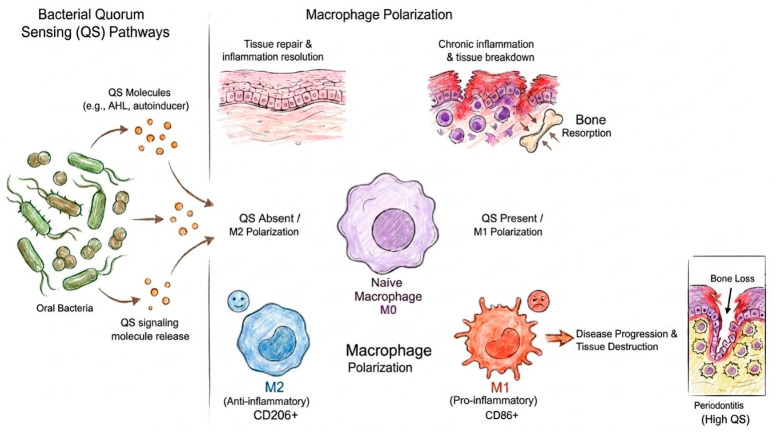
Influence of bacterial quorum sensing on macrophage polarisation and periodontal tissue outcomes. Left panel: without QS signals, tissue homeostasis is maintained [“Summary” heading removed]. Central panel: naïve macrophages (M0) are depicted at the bottom/middle of the figure. Under QS-driven conditions, M0 macrophages polarise toward the M1 phenotype (arrow from M0 to M1); it is M1 macrophages that sustain chronic inflammation and drive tissue destruction through IL-1β, TNF-α, and MMP release. The arrow previously shown from naïve macrophage directly to chronic inflammation has been removed, as M0 macrophages do not contribute to inflammation. M2 polarisation (reparative phenotype) is suppressed when QS signalling is active. Right panel: sustained M1 activity leads to progressive alveolar bone loss and attachment loss. Polarisation labels (M0, M1, M2) are placed adjacent to macrophage illustrations in the central region. Created with FigureLab.

QS pathways also influence immune cell behaviours and polarisation. In periodontitis, macrophage polarisation toward the pro-inflammatory M1 phenotype is associated with greater tissue destruction, while the anti-inflammatory M2 phenotype supports repair and resolution. QS molecules skew macrophages towards M1, sustaining chronic inflammation and the breakdown of periodontal tissues [39,40] (Figure 6).

Neutrophil function is similarly affected. QS-mediated signals can alter chemotaxis, the oxidative burst, and the release of neutrophil extracellular traps (NETs)—processes whose dysregulation is a hallmark of periodontal disease. The net result is impaired bacterial clearance and increased collateral tissue damage. Epithelial barrier function is also compromised: QS molecules modify junctional proteins, opening routes for bacterial invasion and deeper tissue colonisation [41,42].

Because QS molecules act on both bacteria and host cells, quorum quenching offers dual benefit: reducing bacterial virulence and biofilm formation while also dampening excessive host inflammation. This positions QS-targeted therapy within the broader logic of host modulation in periodontics. The receptors, signalling pathways, and downstream effectors that bridge QS molecules to host cells are not yet fully mapped. Defining them more precisely is an important step towards interventions that act simultaneously on microbial virulence and on the host response [20,43].

### 2.4. Quorum Quenching Strategies

Rising rates of antimicrobial resistance have refocused attention on therapies that target bacterial pathogenicity rather than bacterial growth. Anti-virulence approaches attenuate the harmful behaviours of pathogens while leaving cell viability largely intact. Among these, interference with QS—the system that coordinates virulence factor expression and biofilm formation—has been one of the most productive lines of investigation [44,45].

Quorum quenching (QQ) is the disruption of QS signalling, and so the disruption of coordinated bacterial pathogenicity. One approach targets the transcriptional regulators that control virulence gene expression; blocking them suppresses the production of virulence factors that drive disease [46]. Inhibition of homoserine lactone (HSL)-dependent transcription in *Pseudomonas aeruginosa*, for example, lowers expression of multiple virulence-associated genes. Because these strategies interfere with pathogenic mechanisms rather than with growth itself, they exert weaker selective pressure for resistance than conventional antibiotics [47,48].

The same resistance pressures have pushed the search for QS-disrupting molecules, with growing attention to natural compounds and secondary metabolites. Plant-derived flavonoids and phenolics, for instance, inhibit QS signalling across a range of bacteria including *Pseudomonas aeruginosa* (*P. aeruginosa*) and several other pathogens. Such compounds interfere with signal recognition, receptor binding, or transcriptional activation, lowering virulence factor production and overall pathogenicity [49,50].

A more direct strategy uses small molecules to block bacterial chemical communication. In Gram-positive bacteria, the QS signals are typically autoinducing peptides (AIPs) detected by membrane-associated receptors; peptide mimics of these signals can act as competitive antagonists, blocking receptor activation and breaking the QS cascade. Peptide antagonists targeting the agr system in Staphylococcus aureus have inhibited QS-mediated virulence both in vitro and in experimental infections, and analogous approaches have been proposed for LuxR-type transcriptional regulators in Gram-negative bacteria [51,52,53].

A complementary strategy is enzymatic degradation of the signals themselves. Lactonases, acylases, and oxidoreductases inactivate the AHL signalling molecules of Gram-negative bacteria before they reach the concentration needed to trigger coordinated gene expression, severing communication between cells [54].

Table 1 summarises the principal classes of QS inhibitors and their proposed mechanisms of action.

## 3. The Effects of Quorum Sensing Quenching on Periodontal Disease

Because periodontal infections are biofilm diseases, therapeutic interest in disrupting bacterial communication has grown alongside the molecular characterisation of QS itself. In dental plaque, QS coordinates interspecies cooperation, virulence expression, and biofilm maturation; interrupting it offers a way to attenuate pathogenic synergy and shift the microbial balance back towards homeostasis [58]. The LuxS/AI-2 system, which underwrites much of the interspecies signalling in plaque, has been particularly well-studied: inhibition reduces biofilm formation and disorganises virulence factor expression in *P. gingivalis*, *F. nucleatum*, and *A. actinomycetemcomitans* [59,60,61]. In current clinical practice, periodontal treatment follows the European Federation of Periodontology (EFP) S3 guideline stepwise algorithm, which prioritises full-mouth subgingival instrumentation before any adjunctive pharmacological intervention [62]. Quorum quenching agents fit logically into this framework as Step 2 or Step 3 adjuncts—applied after adequate mechanical debridement—rather than as first-line substitutes for it.

Because periodontal biofilms are by definition multi-species, targeting AI-2 signalling disrupts the cooperative interactions that make the community persistent and pathogenic. Inhibiting these signals interferes with adhesion, extracellular matrix production, and virulence determinant expression. Crucially, it does so without eliminating the bacteria themselves, preserving the broader ecological balance and exerting weaker selective pressure for resistance [63,64].

Natural compounds and small-molecule inhibitors have also been tested against oral organisms. Plant-derived flavonoids and phenolic metabolites inhibit QS and reduce biofilm formation in experimental models of periodontal pathogens, while enzymatic degradation of signalling molecules offers an alternative route to attenuating bacterial communication and the coordinated virulence it sustains [50,65,66].

Clinical translation of quorum quenching in periodontal therapy remains at an early stage. Current evidence does not yet support its use as a standalone protocol; rather, the available data support its investigation as an adjunct to scaling and root planning within the EFP S3 stepwise treatment framework [62]. Most of the supporting evidence comes from in vitro or simple experimental models, and the complexity of the polymicrobial oral biofilm makes it difficult to predict how a given inhibitor will behave in vivo. The ecological consequences of interfering with bacterial communication in the oral microbiome also need careful evaluation. Priorities for the field are identifying QS inhibitors that are both effective and ecologically safe, characterising their activity in multispecies biofilm models, and testing their value as adjuncts to mechanical and host-modulatory therapy [67,68].

### 3.1. Antibiotics as Quorum Sensing Inhibitors

Several conventional antibiotics interfere with QS in addition to their direct antimicrobial action; at sub-inhibitory concentrations they suppress QS-regulated virulence factor production without arresting bacterial growth, attenuating pathogenicity rather than killing cells. Quinolone derivatives and macrolides were among the first agents shown to reduce QS-regulated virulence in *Pseudomonas aeruginosa*—the long-standing model organism for bacterial communication [69,70].

Some antibiotics intersect QS at the level of the signalling molecules themselves or their biosynthetic pathways. Sub-inhibitory concentrations of novobiocin, a DNA synthesis inhibitor, disrupt AI-2 signalling and bioluminescence in *Vibrio harveyi*, showing that the metabolic effects of an antibiotic can change QS-dependent phenotypes independently of growth inhibition [71,72].

Among the tetracyclines, doxycycline inhibits the production of virulence-associated enzymes—gelatinase and serine proteases—in *Enterococcus faecalis* (*E. faecalis*). These enzymes are under the control of the fsr QS system, and their suppression has been demonstrated using bioluminescent reporter assays [73]. Because doxycycline acts both as an antimicrobial and as a QS inhibitor, it cannot be considered a pure quenching agent. Its clinical relevance to periodontology is nonetheless considerable: *E. faecalis* is associated with both endodontic and periodontal infection, and doxycycline is already a standard adjunct in periodontal therapy [73,74]. These observations raise the possibility that doxycycline modulates QS pathways in vivo as part of its therapeutic effect.

**Figure 7:**
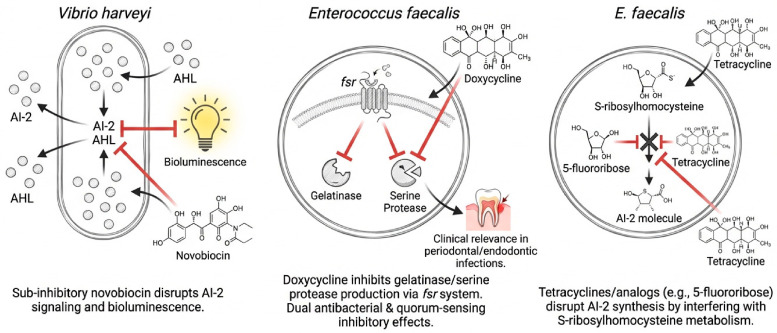
Illustration of antibiotic-mediated quorum-sensing inhibition in bacterial pathogens. Sub-inhibitory concentrations of novobiocin disrupt AI-2 signalling and bioluminescence in *Vibrio harveyi*. In *Enterococcus faecalis*, doxycycline inhibits the production of virulence-associated enzymes such as gelatinase and serine protease regulated by the fsr quorum-sensing system. Tetracycline and related analogues interfere with S-ribosyl homocysteine metabolism, affecting the synthesis of the AI-2 signalling molecule. These mechanisms highlight the potential of certain antibiotics to attenuate bacterial virulence by targeting quorum-sensing pathways rather than bacterial viability Figure created by the authors using the FigureLab AI web-based platform (https://chat.figurelabs.ai).

Some tetracyclines also intersect AI-2 biosynthesis itself by interfering with S-ribosyl homocysteine metabolism, the upstream precursor of AI-2. Analogue compounds such as 5-fluororibose disrupt QS-dependent gene regulation in *E. faecalis* through competitive inhibition and metabolic pathway interference [75,76] (Figure 7).

More recently, computationally designed QS inhibitors have been developed to target specific metabolic steps in signal production without affecting growth. By suppressing virulence-related processes while sparing commensal microbiota, such agents are candidates for combination with conventional antimicrobials and with host-directed therapy [35,77,78].

### 3.2. Natural Compounds as Quorum-Sensing Quenchers

Natural products are attractive candidate QS inhibitors because they show biological activity across a wide range of targets while maintaining relatively low host toxicity. Combined with the broader clinical interest in phytotherapeutics, this has driven sustained research into plant-derived compounds that interfere with bacterial communication, with potential applications across infectious disease and periodontal disease in particular [50,79].

The best-characterised example is the green-tea catechin epigallocatechin gallate (EGCG), which inhibits QS-regulated phenotypes in *P. aeruginosa*. EGCG appears to act on the LasR transcriptional regulator by reducing the availability of cognate autoinducers rather than by inhibiting receptor synthesis itself [55,56]—a relatively unusual mechanism, since most QS inhibitors target signal generation.

A second appeal of plant-derived QQ agents is that they may work with the host immune response. By reducing virulence factor expression, they render bacteria more susceptible to phagocytosis and other host clearance mechanisms. Garlic extract and azithromycin, for example, both improve clearance of *Vibrio harveyi* from oyster hemolymph by interfering with QS [80,81].

Disruption of QS-regulated pathways can also raise the susceptibility of bacteria to immune-mediated killing. In Hemophilus influenzae, antisense inhibition of an essential iron-acquisition outer membrane protein increased sensitivity to human neutrophils and to antimicrobial peptides such as cathelicidin [82,83]. Natural products can thus deliver selective inhibition of virulence gene expression, as in studies where flavonoids reduced extracellular protein production in *Streptococcus mutans* [84].

Natural compounds with documented QS- and virulence-modulating activity are listed in Table 2.

### 3.3. Synthetic Molecules for Quorum Sensing Inhibition

Synthetic molecules designed to disrupt QS provide another route to controlling bacterial virulence. Many such compounds are chemical derivatives of natural products, modified to improve activity, specificity, or stability [88]. The ajoene analogues are a useful example: derivatives of ajoene, a sulphur-containing molecule in garlic originally noted for its anticoagulant properties and later for anti-biofilm activity against Staphylococcus aureus and Staphylococcus epidermidis. Synthetic ajoene analogues show greater anti-biofilm activity and lower toxicity than the parent compound [57,59].

A more refined approach is the rational design of synthetic analogues that mimic natural QS signals. By tuning the chemical structure of a natural ligand or designing molecules that engage QS receptors directly, it is possible to generate inhibitors with higher binding affinity and improved specificity. Such compounds offer practical advantages: predictable activity, less ecological disruption, and tractable pharmaceutical scale-up [23,87]. The value of peptide-based QSI is illustrated by RIP (RNAIII-Inhibiting Peptide), a heptapeptide that competitively antagonises the Staphylococcus aureus AGR (accessory gene regulator) quorum sensing system, thereby suppressing staphylococcal virulence factor production and enhancing antibiotic efficacy in combination regimens [89]. An analogous target exists in the oral Gram-positive context: *Streptococcus mutans* uses the Competence-Stimulating Peptide (CSP)/ComDE two-component system—a signal peptide detected by a membrane-bound histidine kinase (ComD) feeding into the response regulator ComE—to regulate biofilm formation, bacteriocin production, and genetic competence [90]. Synthetic analogues of CSP that act as competitive antagonists of ComD can disrupt biofilm formation in *S. mutans* without bactericidal activity. Compared with the S. aureus AGR system, the CSP/ComDE chemistry differs: AGR uses a thiolactone-modified AIP, while CSP is a linear peptide, making the structure-activity relationships distinct and requiring separate rational design programmes. Delivery to the periodontal pocket introduces additional feasibility constraints: the sulcular fluid environment and subgingival biofilm architecture limit peptide diffusion and stability, favouring formulations with mucoadhesive or encapsulated delivery vehicles to maintain local concentration above the inhibitory threshold.

### 3.4. Artificial Intelligence in Quorum Sensing Drug Discovery

**Figure 8:**
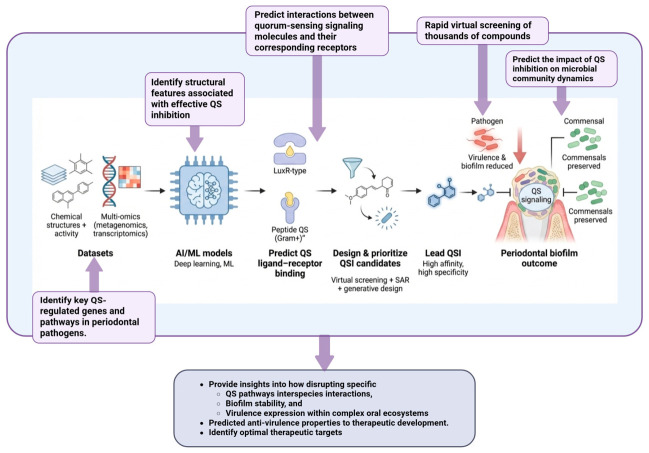
AI-driven framework for targeting quorum sensing (QS) in periodontal biofilms. Schematic illustration of an integrated artificial intelligence (AI) and multi-omics approach for the identification and modulation of quorum-sensing pathways in periodontal disease. Multi-omics datasets and chemical structure libraries are analysed using AI/machine learning models to identify structural features and predict interactions between QS signalling molecules (e.g., AI-2, peptide-based signals) and their corresponding receptors. These predictions enable the rational design and virtual screening of candidate quorum-sensing inhibitors (QSIs), facilitating rapid identification of lead compounds with high specificity and efficacy. The impact of QS inhibition is then evaluated at the biofilm level, aiming to suppress pathogenic virulence while preserving beneficial commensal microorganisms and maintaining microbial homeostasis. This precision-based strategy provides insights into biofilm dynamics, supports drug discovery, and identifies optimal therapeutic targets for periodontal disease management. Created in BioRender. Babiker, R. (2026) https://BioRender.com/8jp2ial (accessed on 1 May 2026).

Artificial intelligence (AI) and computational biology have accelerated the search for QS inhibitors (Figure 8). Conventional QS drug discovery is slow and constrained by the combinatorial complexity of bacterial communication networks; machine-learning–based approaches can shorten this timeline and help identify compounds that target QS pathways while sparing commensal microbiota [91].

Machine-learning and deep-learning models can be used to predict interactions between QS signals and their cognate receptors—LuxR-type transcriptional regulators in Gram-negatives, peptide-detection systems in Gram-positives. Trained on large chemical and bioactivity datasets, these models identify structural features associated with QS inhibition and propose candidate molecules with improved binding affinity and specificity. They also enable rapid virtual screening of thousands of compounds, cutting the time and cost of experimental hit-finding [92,93].

Beyond ligand–receptor modelling, AI methods can simulate polymicrobial biofilm environments and predict how QS inhibition propagates through community-level dynamics. Such models incorporate microbiome composition data to forecast effects on interspecies interactions, biofilm stability, and virulence expression. The approach is especially valuable in periodontal disease, where polymicrobial synergy is central to pathogenesis [94,95].

AI also assists the rational refinement of existing natural and synthetic QQ molecules. Structure–activity relationship (SAR) modelling can guide optimisation of flavonoids, phenolics, and peptide analogues for stability, bioavailability, and target specificity. Generative models go further, designing entirely novel chemical scaffolds with predicted anti-virulence activity [96].

A complementary application pairs AI with omics—metagenomics and transcriptomics—to pinpoint QS-regulated genes and pathways in periodontal pathogens. Linking gene expression to QS activity reveals the critical nodes in these communication networks and helps prioritise them as therapeutic targets. The systems-level view supports the design of interventions that suppress pathogenic signalling while preserving beneficial microbial functions [97,98].

These advances are not without limitations. Predictive accuracy depends on the quality and diversity of the training data, and the available datasets for many oral organisms remain limited. In silico predictions must still be validated experimentally and clinically. Ethical and regulatory frameworks for AI-driven drug discovery are themselves at an early stage of development. Even so, the addition of AI to QS research is a meaningful change in how anti-virulence agents can be designed: data-driven hypotheses, faster iteration, and earlier triage of candidates against periodontal pathogens.

### 3.5. Efficacy of Quorum Sensing Quenching

Animal models—principally mouse and rat ligature systems—have clarified how interactions among periodontal pathogens drive disease and how disrupting those interactions might lower pathogenicity [98]. *Prevotella intermedia* (*P. intermedia*) on its own causes little periodontal pathology, but co-infection with *Porphyromonas gingivalis* in mice produces marked alveolar bone loss—evidence of microbial synergy at the level of pathogenesis [99,100,101]. By extension, disrupting the communication that supports such synergy is an attractive strategy for slowing periodontal disease.

In vitro work on periodontal pathogens has shown that QS inhibitors interfere with virulence and biofilm formation. Garlic-derived compounds, for example, inhibit biofilm formation and suppress the production of virulence-associated metabolites in *P. gingivalis*. Plant-derived extracts and phytochemicals more broadly disrupt biofilm maturation and antagonise key periodontal pathogens. The signal is encouraging, but the data are almost entirely from laboratory systems; clinical efficacy cannot be established without validation in animal models and human trials [20,102,103].

### 3.6. Safety Considerations of Quorum-Sensing Inhibitors

One argument for the safety of QS inhibitors is that they attenuate virulence rather than kill bacteria [101]. Initial experimental data are consistent with this: many candidate inhibitors show low host-cell toxicity while still suppressing bacterial communication. Ethanolic extracts of garlic and purple coneflower and certain plant essential oils inhibit AI-2 production by Clostridium perfringens at sub-MIC concentrations, without affecting bacterial growth. Comparable activity has been reported for green tea and its polyphenols [104,105].

Collectively, these data suggest that QQ agents reduce bacterial virulence without major disruption of the wider microbial community. Sub-MIC essential oils have similarly been shown to suppress virulence-associated traits without arresting growth. The data, however, are predominantly in vitro, and a more thorough safety profile is needed before any of these compounds can be advanced to clinical use [106,107].

Formal safety assessment—toxicity profiling, pharmacokinetics, and screening for systemic effects—is a prerequisite to human trials [107]. Because these compounds operate on fundamental bacterial signalling pathways, their indirect effects on host–microbiome equilibrium also require careful assessment during development.

#### Challenges and Future Directions: Toward Precision Periodontal Therapy

Several factors limit the clinical translation of QS-based strategies in periodontology. The most obvious is the scarcity of well-designed human trials; most current evidence comes from in vitro and preclinical work that does not fully capture in vivo disease. The natural history of periodontitis itself adds difficulty: the disease is heterogeneous and episodic, with fluctuating activity that makes consistent clinical endpoints hard to define. Surrogate biomarkers are often used as a workaround, but they themselves require validation.

A second issue is the ecological complexity of the oral microbiome. QS regulates pathogenic virulence but also supports homeostatic interspecies cooperation and metabolic cross-feeding. Disrupting these signals can therefore reshape community structure in unintended ways. Broad, non-selective interference with QS would affect commensals as well as pathogens, with the paradoxical risk of inducing dysbiosis or opening niches to opportunistic colonisers. To make this ecological framing operationally useful, three selectivity strategies merit explicit development. First, rather than broad LuxS/AI-2 disruption, inhibitors could be directed specifically at taxa in which AI-2 signalling is coupled to defined virulence circuits—for example, targeting the *P. gingivalis* LuxS/AI-2 system that drives gingipain and FimA expression, while sparing AI-2-dependent cooperative metabolism in commensal streptococci that do not carry these virulence determinants.

This requires species-resolved dissection of which organisms in the plaque community use AI-2 for pathogenic versus homeostatic purposes—a tractable task with current single-cell transcriptomics and imaging-based interspecies dynamic approaches.

Second, receptor-level antagonists with narrow species-binding profiles are preferable to broad signal-degrading enzymes. Peptide mimics designed against ComDE receptors of *S. mutans*, or against the specific LuxR-type regulators of *A. actinomycetemcomitans* that control leukotoxin expression, would leave other QS networks intact. Structure-guided design of species-specific receptor antagonists—informed by comparative structural biology of homologous QS receptors across oral taxa—is a realistic near-term objective for the field. Third, delivery engineering is inseparable from selectivity. Subgingival delivery systems designed for pocket retention—mucoadhesive gels, PLGA microspheres, or lipid nanoparticles with controlled release kinetics—concentrate the active compound within the 1–3 mm depth of the periodontal pocket and minimise systemic diffusion and perturbation of supragingival commensals. Targeting deep pockets (≥4 mm) rather than shallow sites further narrows exposure to the dysbiotic subgingival community where virulence-associated QS activity is most relevant.

Microbial adaptability is a third constraint. Bacteria can mount compensatory responses—activation of alternative signalling pathways or modification of receptor systems—that bypass QS inhibition. Over time, such responses may erode the long-term effectiveness of QQ strategies. The concept of functional or “silent” dysbiosis adds another layer of difficulty: a community may look compositionally stable on standard sequencing while quietly sustaining pathogenic activity that conventional analyses miss.

These caveats notwithstanding, the growing understanding of QS in polymicrobial biofilms provides a foundation for precision periodontal therapy. Periodontal disease is heterogeneous in microbial composition, host immune response, and genetic susceptibility, and this heterogeneity is itself an argument for individualised treatment. QS pathways are well-suited to that logic: they are biological targets that can be modulated in a patient-specific way.

QS activity may also have value as a functional biomarker for disease stratification. Distinct communication profiles—reflecting which pathogens and which signalling systems dominate—may underlie clinical phenotypes and differential treatment responses. Combining QS-related markers with clinical parameters could help identify patient subgroups with different microbial and inflammatory dynamics, and so support more targeted interventions.

Omics technologies—metagenomics, transcriptomics, and metabolomics—provide the tools to characterise microbial communication networks at both compositional and functional levels. Applied to oral samples, they could allow for near-real-time assessment of QS activity in the periodontal ecosystem and guide selective application of QS inhibitors against specific signalling pathways, minimising disruption of beneficial interactions while attenuating pathogenic virulence. A prerequisite for meaningful cross-study comparison is the establishment of shared, validated experimental standards for QS research in periodontology. At present, studies differ substantially in the bacterial strains used, the outcome measures reported, and the biofilm models employed, making it difficult to determine whether conflicting results reflect genuine biological differences or methodological variation. Two priorities stand out. First, validated AI-2 reporter systems—such as the V. harveyi BB170 bioluminescence reporter or recombinant LuxS-deficient strains reintroduced with reporter constructs—should be adopted as reference standards for measuring AI-2 activity in oral polymicrobial experiments, allowing AI-2 quantification to be directly comparable across laboratories. Second, the field would benefit from a community-agreed standardised multispecies oral biofilm model incorporating defined proportions of representative early colonisers (*Streptococcus oralis*, *Actinomyces naeslundii*), bridging species (*Fusobacterium nucleatum*), and late pathogens (*P. gingivalis*, *T. denticola*, *A. actinomycetemcomitans*) on hydroxyapatite or dentine discs, with standardised growth conditions and endpoint measures. Such a model—analogous to the standardised datasets that have driven progress in AI-assisted caries radiographic detection—would allow QS inhibitor efficacy data to be accumulated on a common biological reference point and would substantially accelerate translation of in vitro findings toward clinical evaluation.

Combining QS-targeted therapy with mechanical debridement and host modulation may improve treatment outcomes while reducing reliance on broad-spectrum antimicrobials. Realising this in practice will require reliable biomarkers, standardised diagnostic methods, and rigorous clinical validation. The wider shift towards precision-based modulation of microbial behaviour and host response is a credible route to more predictable, longer-term outcomes in periodontal disease. Among the inhibitor classes covered in this review, sub-inhibitory doxycycline is the most immediately applicable: it has regulatory approval for periodontal use, a well-characterised pharmacokinetic profile, and clinical trial data supporting its adjunctive use alongside mechanical debridement.

Plant-derived QS inhibitors such as EGCG and garlic-derived compounds offer a favourable safety margin but have not yet been tested in adequately powered clinical trials in periodontitis patients. Synthetic inhibitors carry the most precise mechanism of action but require full pre-clinical and clinical development before they can reach patients. For the near term, optimising doxycycline delivery—for example via sustained-release PLGA microspheres directly into the periodontal pocket—is the most realistic path to clinical use; for the medium term, AI-guided screening of natural and synthetic QS inhibitor libraries is the approach most likely to generate viable new candidates.

## 4. Conclusions

Periodontal disease is a polymicrobial condition in which microbial dysbiosis and host inflammatory responses combine to destroy tooth-supporting tissues. Within these communities, quorum sensing (QS) is a central regulator: it coordinates virulence factor production, biofilm formation, and the interspecies interactions through which periodontal pathogens persist and inflict damage in the oral environment.

Quorum quenching (QQ) has accordingly emerged as a coherent anti-virulence strategy. Across the QS inhibitor classes—sub-inhibitory antibiotics, plant-derived natural products, and rationally designed synthetic molecules—agents disrupt microbial signalling and reduce biofilm formation and virulence expression in pathogenic bacteria. Experimental work on periodontal pathogens specifically suggests that QS interference can attenuate the microbial cooperation and pathogenic synergy that sustain dental plaque biofilms.

Clinical application of QS inhibition in periodontal therapy remains, however, at an early stage. Most data are from in vitro and animal studies, and the ecological interactions of the polymicrobial oral biofilm make translation more difficult than for single-pathogen infections. Comprehensive safety profiling and well-powered clinical trials are needed to establish the efficacy, specificity, and long-term effects of QQ agents in patients.

Productive future directions include further dissection of the molecular mechanisms governing bacterial communication in periodontal biofilms, identification of QS inhibitors that are both effective and ecologically tolerable, and evaluation of these agents in multispecies biofilm models and in clinical settings. By targeting virulence rather than viability, QQ offers an adjunct to conventional periodontal therapy with a lower theoretical risk of selecting for antimicrobial resistance—an attractive proposition as the field moves towards more biology-led, precision-oriented management of periodontal disease.

## Figures and Tables

**Table 1:** Classes of quorum-sensing inhibitors and their mechanisms relevant to periodontal pathogens.

Class of Quorum-Sensing Inhibitor	Mechanism of Action	Example Compounds	Target Organisms/Relevance	Key Effect	References
Enzymatic quorum-quenching agents	Degradation or modification of signalling molecules (e.g., AHLs, AI-2)	Lactonases, acylases	Various Gram-negative bacteria including oral biofilm species	Disruption of bacterial communication and reduced virulence expression	[54]
Antibiotics with QS inhibitory activity	Interference with QS signalling pathways at sub-inhibitory concentrations	Macrolides, doxycycline, novobiocin	*Pseudomonas aeruginosa*, *Enterococcus faecalis*, oral pathogens	Suppression of virulence factor production and biofilm formation	[55]
Natural quorum-sensing inhibitors	Inhibition of signal synthesis or receptor activity	EGCG (green tea), garlic extract, flavonoids	*P. gingivalis*, *S. mutans*, *P. aeruginosa*	Reduced biofilm formation and virulence gene expression	[56]
Synthetic QS inhibitors	Rationally designed molecules targeting signal receptors or signalling enzymes	Ajoene analogues, AHL analogues	Various pathogenic bacteria	Competitive inhibition of QS signalling pathways	[57]
Signal analogue antagonists	Structural mimicry of autoinducer molecules blocking receptor binding	Synthetic peptide antagonists	Gram-positive bacteria (agr system)	Inhibition of QS-regulated virulence	[58]

**Table 2:** Natural compounds reported to interfere with quorum sensing and virulence-associated phenotypes.

Natural Compound/Source	Target Organism	Proposed Quorum Sensing Target	Observed Effect	Reference
Epigallocatechin gallate (EGCG)—Green tea (*Camellia sinensis*)	*Pseudomonas aeruginosa*	Interference with LasR signalling pathway and autoinducer availability	Reduced QS-regulated virulence phenotypes	[54]
Garlic extract (*Allium sativum*)	*Vibrio harveyi*	Disruption of QS signalling pathways	Enhanced pathogen clearance and reduced virulence	[85]
Plant essential oils and phytochemicals	Various bacterial pathogens	Inhibition of AI-2 signalling	Suppression of QS activity at sub-MIC concentrations	[86]
Flavonoids (plant-derived compounds)	*Streptococcus mutans*	Inhibition of QS-regulated extracellular protein synthesis	Reduced virulence-associated protein production	[87]

## Data Availability

No new data were created or analysed in this study. Data sharing is not applicable to this article.
